# Dual antiplatelet therapy up to the time of non-elective coronary artery bypass grafting with prophylactic platelet transfusion: is it safe?

**DOI:** 10.1186/s13019-019-1028-2

**Published:** 2019-11-27

**Authors:** Fida Charif, Righab Hamdan, Genane Youness, Ali El Zein, Mohamad Issa, Yehya Jassar, Mahmoud Younes, Mohamad Saab

**Affiliations:** 1 Division of Pulmonary Medicine, Beirut Cardiac Institute, Beirut, Lebanon; 2 Division of Cardiology Medicine, Beirut Cardiac Institute, Beirut, Lebanon; 3 Department of statistics, ISSAE, Cnam, Beirut, Lebanon; 4 Department of Anesthesiology, Beirut Cardiac Institute, Beirut, Lebanon; 5 Department of Cardiovascular surgery, Beirut Cardiac Institute, Beirut, Lebanon; 6 Medical research center, Beirut Cardiac Institute, Rassoul Aazam Hospital, Beirut, Lebanon

**Keywords:** Aspirine, Clopidogrel, CABG, ACS, Platelet transfusion

## Abstract

**Background:**

Guidelines suggest that patients discontinue Clopidogrel at least 5 days prior to coronary artery bypass grafting (CABG). Those with acute coronary syndrome (ACS) are at high risk for myocardial infarction (MI) if not treated with dual antiplatelet therapy (DAPT). We sought to assess pre and post-operative outcomes of patients maintained on Clopidogrel and aspirin up to the time of surgery and compare them with those on aspirin alone.

**Methods:**

From the cardiac surgery database, 240 patients were retrospectively registered between January and May 2017. There were 126 patients with ACS who underwent CABG on DAPT (Clopidogrel group [CG]) and 114 patients who underwent elective CABG on aspirin alone (control). The CG received intraoperative prophylactic platelet transfusion (PPT). Demographics, comorbidities, and laboratory data were prospectively entered at the time of surgery and were subsequently retrieved for analysis. Per and postoperative findings were identified and compared between both groups.

**Results:**

The cohort consisted of 240 patients (mean age 61 years, 81.3% were male, SD ± 9.58). Patients in the CG were younger (Median 57 vs. 63, *P*-value 0.001), and with male predominance (86% versus 75%, *P*-value 0.028). In addition, they had less prevalence for diabetes and renal failure as compared to control (*P*-values 0.003, and 0.005, respectively). There were no significant differences between both groups in number of vessels grafts, duration of on-pump and aortic clamp. Hematologic laboratory data had also similar baseline values. The CG had similar bleeding rate, redo surgery and in-hospital death (*P*-values non-significant), however more infection and total hospital stay as compared to control (*p*-values 0.048 and 0.001).

**Conclusion:**

Patients who are at increased risk for MI can be maintained on DAPT up to the time of CABG because surgery is safe when patients are offered PPT.

## Introduction

Many patients with acute coronary syndrome (ACS) and severe multi-vessel disease are offered non-elective coronary artery bypass grafting (CABG) within a short period of time since their last episode of chest pain [1]. The use of adenosine diphosphate antagonists in the preoperative period carries both benefits and risks. Clopidogrel has been frequently used in the management of ACS. It decreases the risk of new ischemic events in high-risk patients awaiting surgery, but on the other hand, it might increase the risk for post-operative bleeding [2, 3]. The American Heart Association/American College of Cardiology Foundation and the Society of Thoracic Surgeons recommend against continuation, and advise to stop Clopidogrel 5 to 7 days prior to surgery [4, 5]. In addition, they advise for platelet transfusion in high-risk patients for bleeding prevention. However, there have been conflicting recommendations for platelet transfusion at the time of surgery. According to The British Society of Hematology (BSH) [6], the role of prophylactic platelet transfusion to reverse the effect of antiplatelet therapy is unclear. Our retrospective study aims at assessing the effect of exposure to Clopidogrel and aspirin within 5 days of surgery on post-operative bleeding, reoperation, in-hospital length of stay, mortality, and transfusion requirements, and compare the outcomes with patients who underwent CABG on aspirin alone.

## Methods

This is a retrospective analysis, representing the routine practice in our center. From the cardiac surgical unit database at Beirut Cardiac Institute, a total of 240 consecutive patients were retrospectively identified between January 2017 and May 2017. Of them, 126 patients with ACS who underwent urgent or emergent isolated CABG and received Clopidogrel (within 5 days of surgery) and aspirin up to the day of the surgery, were assigned to a Clopidogrel group (CG), and compared to 114 patients who underwent elective CABG on aspirin alone up to the day of surgery, as a control group.

All surgical procedures were performed via a median sternotomy approach using cardiopulmonary bypass and cardioplegic arrest with cristalloid solution and cold water placed in the pericardial cavity during the clamp time.

Harvest of both internal thoracic arteries was performed in a skeletonized fashion, saphenous vein grafts were added in most of the patients.

All patients received 300 IU/Kg of intravenous unfractionated heparin before pump initiation, and 1 g of intravenous tranexamic acid while on-pump, as per our protocol. Patients in the CG received prophylactic 1 pool of platelets concentrate (8 units) early after discontinuation of the pump while in the operating room. Both groups were operated by the same surgical team.

Red blood cells transfusion was based on the patient’s clinical condition rather than on a fixed hemoglobin value. However we followed mainly these rules:

- Hemoglobin less than 8 mg/dl for stable asymptomatic patients, aged less than 65 years, with mixed venous oxygen saturation (SVO2) above 65%.
- Hemoglobin between 8 and 10 mg/dl for symptomatic patients aged more than 65 years and who have an ejection fraction less than 50% and SVO2 less than 65%.

Indication of Fresh Frozen Plasma (FFP) transfusion was and increased in perioperative blood loss with prolonged international normalized ratio (INR) more than 1.5.

Indications for platelet transfusion was thrombocytopenia less than 50 × 10^9^/L and/or chest tube drainage more than 300 cc/hour.

Demographics, comorbidities, laboratory data and other confounding factors were prospectively entered at the time of surgery and were subsequently retrieved for analysis.

### End points

There were 3 co-primary endpoints: major bleeding, reoperation rate for bleeding, in hospital mortality and blood products transfusion requirement. Secondary endpoints were: 1) length of stay in the intensive care unit (ICU) and total hospital stay; 2) in-hospital infection; 3) stroke.

### Outcome characteristics

Major bleeding was defined as chest tube drainage > 1500 ml, intracranial bleeding, or cardiac tamponade. Post-operative bleeding was defined as the total amount of chest tube drainage till its removal. Severe post-operative bleeding was defined as post-operative chest tube drainage more than 300 ml/hour for 3 consecutive hours. Transfusion requirements were identified as the total amount of all blood product transfusions including unit packed red blood cells (UPC), fresh frozen plasma (FFP) and platelets concentrates during intraoperative and postoperative periods till patient’s discharge. Stroke was defined as any hemorrhagic or ischemic events.

### Statistical analysis

Continuous variables were described using median. Categorical variables were described as percentages. The chi-square test was used for categorical variables. The Mann –Whitney U test was used for non parametric continuous variables. *P*-value of < 0.05 was considered statistically significant.

## Results

### Baseline characteristics and preoperative hematologic variables

The cohort consisted of 240 patients (mean age 61 years, 81.3% were male, SD ± 9.58). All the patients in the Clopidogrel group were in acute coronary syndrome, 70% of these patients had positive troponin and 60% had critical left main lesion, whereas only 15 % of the control group had left main involvement, and none of the control patients had positive troponin. In addition patients assigned to the CG were younger (age 57 vs. 63, *P*-value 0.001), and with male predominance (86% vs. 75%, *P*-value 0.028). We found as well less diabetes, dyslipidemia, and renal failure in the CG compared to the control group (*P*-values 0.003, 0.028, and 0.005, respectively). There were no significant differences between both groups in platelets count, hematocrit, international normalized ratio and activated prothrombin time levels and number of vessels grafts. All results are shown in Table 1 laboratory data on admission are shown in Table 2.

**Table 1:** Patients characteristics

Variable	Control group (*n* = 114)		Clopidogrel group (*n* = 126)		*P*-value
Patients characteristics					
Sex, male	86	(75.4)	114	(86.5)	.028^a^
Age	63	(57–70)	58	(52–65)	.001^b^
Body weight	77.5	(68–84)	79	(69–85)	.874^b^
Dyslipidemic	45	(39.5)	33	(26. 6)	.028^a^
Smoker	85	(74. 6)	86	(68.3)	.281^a^
Diabetic	67	(58.8)	50	(39.7)	.003^a^
Hypertensive	73	(64.0)	72	(57. 6)	.276^a^
Renal dysfunction	13	(11.4)	3	(2.4)	.005^a^
Ejection Fraction	59	(50–63)	55.5	(45–63)	.368^b^

^a^χ^2^ test, n (%)

^b^Mann-Whitney independent samples, median + interquartile range

**Table 2:** Laboratory Data on admission

Variable on admission	Control group (*n* = 114)		Clopidogrel group (*n* = 126)		*P*-value
Labs					
Number of vessels grafts	3.21	(3-4)	3	(3-3)	.061
Aortic Clamp (min)	41.5	(35–50)	42	(37–51)	.310
ECC time (min)	73.5	(62–83)	71	(62–83)	.643
PTT	35	(34–36)	37	(34–39)	.000
INR	1	(1-1)	1	(1-1)	.292
Platelets	251.5	(198–289)	243	(203–288)	.390
Hemoglobin	13.7	(12-15)	14	(13-15)	.155
Hematocrit	41	(37–44)	41	(38–44)	.596

*ECC* Extracorporeal circulation

*PTT* Partial thromboplastin time test

*INR* International Normalized Ratio

^a^ χ^2^ test, n (%)

^b^ Mann-Whitney independent samples, median + interquartile range

### Per- and post-operative variables stratified by CG vs. control group

**Fig. 1:**
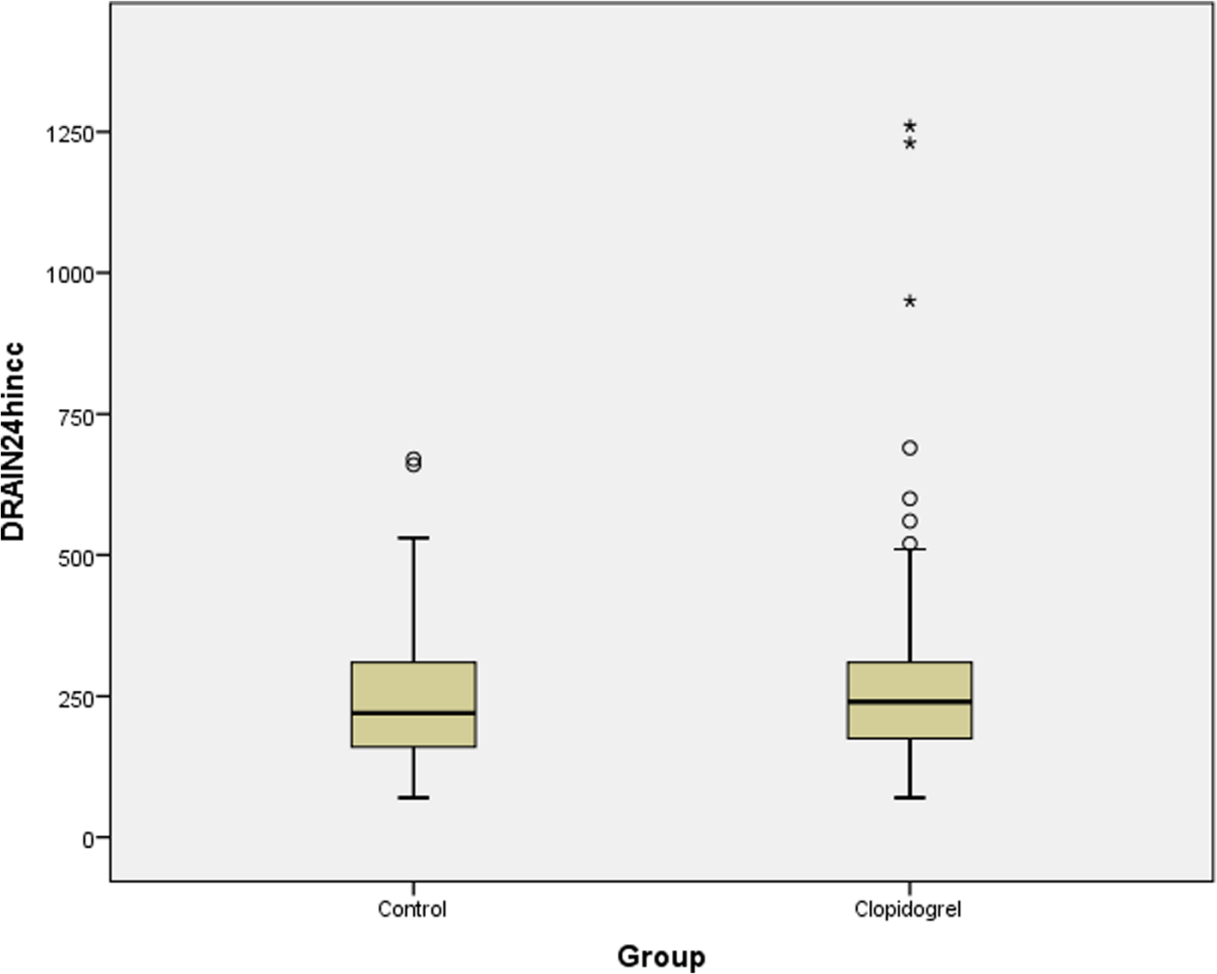
Drain output per 24 h in cc

Patients who were assigned to the CG had similar bleeding rate, (Fig. 1) redo surgery and in-hospital death (non-significant *P*-values), moreover there was no statistical significance regarding the amount of blood products transfusion and ICU stay between both groups. However, patients in the CG had more platelets transfusion (that was mainly attributed to the received preoperative prophylactic pools of platelets as previously described *P*- value 0.001), infection, and total hospital stay as compared to those in the control group (*P*-values 0.048 and 0.001 respectively). Reoperation rate was negligible in the 2 groups as well (*P*-value = 1). All results are shown in Table 3. Multiple linear regression analysis showed that the increased total hospital stay and infection rate seen in the Clopidogrel group was mainly attributed to platelets transfusion (F (5,235) = 603.56,*P*-value< 0.0001 with *R*^*2*^ = 0.93).

**Table 3:** Results

Variable	Control group (*n* = 114)		Clopidogrel group (*n* = 126)		*P*-value
Results					
DRAIN/24 h in cc	220	(160–313)	240	(180–310)	.476^b^
Infection	0	(0.0)	3	(2.4)	.048^a^
Death	2	(1.8)	1	(0.8)	.501^a^
Reoperation	0	(0.0)	1	(0.8)	.256^a^
Stroke	0	(0.0)	0	(0.0)	1
Blood group					
A+	47	(41.2)	43	(34.1)	.026^a^
A-	6	(5.3)	8	(6.3)	
B+	14	(12.3)	23	(18.3)	
B-	1	(0.9)	6	(4.8)	
AB+	9	(7.9)	6	(4.8)	
AB-	1	(0.9)	1	(0.8)	
O+	27	(23.7)	38	(30.2)	
O-	9	(7.9)	1	(0.8)	
Platelets per Op	0	(0–0)	8	(8-8)	.001^b^
UPC per OP	0	(0–0)	0	(0–0)	.005^b^
FFP per Op	0	(0–0)	0	(0–0)	.087^b^
UPC post Op	0	(0–1)	0	(0–0)	.248^b^
FFP post Op	0	(0–0)	0	(0–0)	.575 ^b^
UPC total	0	(0–1)	0	(0–0)	.014^b^
FFP total	00	(0–0)	0	(0–0)	.470^b^
Hemoglobin D1 post Op	11.4	(10-12)	11.4	(11-12)	.755^b^
Hemoglobin D4 post Op	10.856	(10-12)	10.5	(10-11)	.367^b^
Hematocrit D0 post Op	40.45	(37–43)	41.00	(38–44)	.041^b^
Hematocrit D1 post Op	33.4	(3 1-37)	34.05	(32–37)	.980^b^
Hematocrit D4 post Op	32.234	(30–34)	31.4	(30–34)	.410^b^
Platelets D0 post Op	234	(184–277)	245.5	(203–296)	.083^b^
Platelets D4 post Op	220	(180–257)	254	(210–321)	.001^b^
Length ICU/CSU stay(d)	1.00	(1-1)	1	(1–1)	.098^b^
Total hospital stay(d)	5	(5-6)	6	(6–7)	.001^b^
Creatinine baseline	0.915	(1-1)	0.87	(1-1)	.106^b^
Creatinine dc	1.055	(1-1)	1.055	(1-1)	.791^b^

*Op* Operation

*D* Day

*UPC* Unit packet cells

*FFP* Fresh frozen plasma

^a^χ^2^ test, n (%)

^b^Mann-Whitney independent samples, median + interquartile range

## Discussion

Our center performs around one thousands open heart surgeries per year and is one of the most active centers in the region. Our data shows that cardiovascular surgery with sustained Clopidogrel is feasible and the increasing experience of the cardiovascular team makes it safe. However, past research showed conflicting results. In a prospective observational study Ouattara et al. [7] compared the perioperative bleeding rate in patients who underwent first time CABG on aspirin alone with those on Aspirin and Clopidogrel 5 days prior to surgery while receiving prophylactic low dose Aprotinin (a fibrinolysis inhibitor). There were no differences in the perioperative bleeding rate or transfusions requirements between both groups. In another randomized double blind placebo-controlled study [8], 136 patients with ST-segment elevation myocardial infarction (STEMI) requiring CABG during the same hospitalization were assigned to either CG or placebo at the time of fibrinolysis therapy. There was no significant difference in perioperative bleeding rate, with a reduction of 30 days incidence of adverse ischemic events among the CG. Indeed, in a large prospective randomized trial, Ebrahimi et al. [9] showed that administration of Clopidogrel in non STEMI requiring CABG was associated with fewer adverse ischemic events and non-significant increased post-operative bleeding compared to patients who did not receive Clopidogrel. However, a 5-day washout period prior to surgery was considered in all patients in the CG.

On the other hand, a meta-analysis of 34 studies including 22,584 patients [10] showed increased mortality and post-operative bleeding among Clopidogrel-exposed patients who underwent CABG; nevertheless, authors recommended that high-risk ACS patients should proceed with CABG without delay for a Copidogrel-free period. Another meta-analysis [11] of 6,385 ACS patients who required CABG showed that exposure to Clopidogrel within 5 days prior to CABG was associated with increased major bleeding and reoperation, although it showed significantly lower incidence of mortality and ischemic adverse events. The authors suggested that ACS patients, who were subsequently referred for CABG, should wait for a minimum of 5 days washout period to prevent bleeding and reoperation. Another study conducted by Nurozler et al. [12] showed increased bleeding rate and re-exploration for bleeding in patients exposed to Clopidogrel within a week of CABG. The duration of mechanical ventilation and length of stay were also longer compared to control group among those patients. Miceli et al. [13] showed that Clopidogrel within 5 days in combination with aspirin within 2 days of CABG was associated with an increased risk of postoperative myocardial infarction, bleeding and reoperation for bleeding. Likewise, other retrospective case control studies showed that patients who underwent CABG shortly after Clopidogrel exposure had increased risk of re-exploration for bleeding [14–16], and bleeding risk was significantly higher when Aspirin and Clopidogrel were continued up to 2 days prior to surgery [15].

Bleeding in cardiac surgery is whether surgically induced or due to acquired hemostatic defect. Platelet dysfunction due to antiplatelet therapy prior to CABG is the most important hemostatic factor that may lead to bleeding [5, 17]. According to the American Heart Association/American College of Cardiology Foundation, ACS patients on dual antiplatelet therapy (DAPT) requiring non-elective CABG are considered to be at high risk for bleeding [18]. Recommendations provided by the American and European guidelines regarding prophylactic preprocedural platelet transfusion remain conflictual [19–21] and data regarding PPT in cardiac surgery on DAPT is still a matter of debate. In the absence of clear and questionable recommendations regarding this specific high-risk population, our results show the safety of operating on DAPT. Platelets transfusions should be viewed as scarce resource that has benefits and risks. Previously published data regarding this issue showed conflicting results. In an observational case control study [17], patients who proceeded with CABG (intervention group) and received isolated one pool of platelet transfusion early after discontinuation of the pump while in the operating room were compared to those who did not receive platelets. It showed no difference in reexploration, infection, organ failure and ischemic adverse events between groups. However, the intervention group had less chest tube drainage but experienced prolonged ICU stay, mechanical ventilation, need of inotropic medications and blood products transfusion requirement. In a retrospective cohort study, Karkouty et al. [22] showed that platelet transfusion in patients undergoing cardiac surgery was not associated with increased mortality and morbidity. In contrast, in a retrospective analysis of a double-blind placebo versus control study, Spiess et al. [23] showed that platelet transfusion in patients undergoing CABG was associated with increased serious adverse events, like infection, longer hospital stay, requirement of packed red blood cells transfusions, stroke and death compared to those who did not receive platelet transfusion.

Our study showed that prophylactic platelet transfusion in ACS patients requiring CABG while on DAPT up to less than 5 days prior to surgery is safe. There was no difference in the 3 co-primary and secondary endpoints between both groups. Moreover, chest tube drainage per 24 h in the CG and control group were similar (220 vs. 240 ml, *P*-value 0.476), and, compared to what was previously published, the amount was much smaller [1–3, 6–9, 11–14]. This finding was felt to be, though not truly confirmed, secondary to the surgical expertise and the protective effect of the peroperative prophylactic platelet transfusion.

### Strengths and limitations

The timing of Clopidogrel discontinuation was precisely registered, and was less than 5 days prior to surgery, which might have prevented the adverse ischemic events. The amount of pre, intra, and post-operative blood product transfusions were analyzed. All patients were operated by the same surgical team and followed the same blood product transfusion protocol.

Despite those strengths, we recognize four limitations. First, this is a single center, retrospective, observational study. The sample size is relatively small given the short period of data collection. Second, no preoperative antiplatelet activity was done before PPT, because of non availability. Third, we did not compare the outcome to a true placebo group naïve to Clopidogrel and Aspirine, since in our routine practice we perform most of our surgeries without discontinuing Aspirine, nevertheless this might be an additive criterion for the feasibility and safety of open heart surgery without discontinuing any anti-platelet therapy. It would be of major interest to design a prospective study comparing ACS patients operated without delay and without discontinuing dual anti platelet therapy, while receiving platelet transfusion to ACS ptients operated with 5 to 7 days delay after discontinuing dual anti platelet therapy.

## Conclusions

Based on our findings, we suggest that CABG on DAPT is most likely feasible and safe, especially when patients receive prophylactic peroperative platelet transfusion. More randomized controlled trials are needed to fully determine the role of prophylactic platelet transfusions, in urgent or emergent cardiac surgery, in preventing bleeding in patients on DAPT. Waiting for new results, we suggest that ACS patients requiring CABG proceed with surgery without delay for a Clopidogrel-free period.

## Data Availability

Data and material are available upon request, and may be sent as supplementary material if required.

## Abbreviations

ACS: Acute coronary syndrome
BSH: British Society of Hematology
CABG: Coronary artery bypass grafting
CG: Clopidogrel group
DAPT: Dual antiplatelet therapy
FFP: Fresh frozen plasma
ICU: Intensive care unit
MI: Myocardial infarction
PPT: Prophylactic platelet transfusion
STEMI: ST-segment elevation myocardial infarction
UPC: Unit packed red cells
